# AI literacy and attitudes among maternal and child health nurses: a multicenter psychological network analysis of novices and experts in China

**DOI:** 10.3389/fpubh.2026.1786184

**Published:** 2026-06-02

**Authors:** Qin Zeng, Jun Zhu, Cheng Yang, Jiacheng Hu, Xi Huang

**Affiliations:** 1 Department of Pediatrics Nursing, West China Second University Hospital, Sichuan University, Chengdu, China; 2 Key Laboratory of Birth Defects and Related Diseases of Women and Children, Sichuan University, Ministry of Education, Chengdu, China; 3 Department of Pediatrics, National Office for Maternal and Child Health Surveillance of China, National Center for Birth Defect Surveillance of China, West China Second University Hospital, Chengdu, China; 4 Department of Pediatric Intensive Care Unit Nursing, West China Second University Hospital, Sichuan University, Chengdu, China; 5 Department of Nursing, West China School of Nursing, Sichuan University, Chengdu, Sichuan Province, China; 6 Department of Neonatology Nursing, West China Second University Hospital, Sichuan University, Chengdu, China

**Keywords:** AI fear, AI literacy, artificial intelligence, novice to expert, nursing workforce, psychological network analysis, public health education

## Abstract

**Background:**

The successful integration of Artificial Intelligence (AI) in healthcare depends heavily on the literacy and attitudes of the frontline nursing workforce. While psychological barriers like “AI fear” are known, the complex interplay between literacy and attitudes across different levels of expertise remains poorly understood from a systemic perspective.

**Objective:**

This study aimed to utilize psychological network analysis (PNA) to map and compare the AI literacy and attitude cognitive networks of clinical preceptors (experts) and nurse interns (novices) within the maternal and child health (MCH) nursing context.

**Methods:**

A large-scale, multicenter cross-sectional study was conducted across 32 institutions in 26 provinces in China. A total of 1,031 participants (498 clinical preceptors and 533 nurse interns) completed the AI Literacy Scale and the General Attitudes toward AI Scale. Regularized partial correlation networks were estimated to identify core cognitive nodes. The Network Comparison Test (NCT) was employed to evaluate statistical differences in network topology and global strength between the expert and novice groups.

**Results:**

“AI Fear” (specifically technological dread and future anxiety) emerged as the most central and influential node, dominating the cognitive networks of both groups. However, the overall network topology differed significantly between the two groups (*p* = 0.023). Novices exhibited a “high-density, undifferentiated” structure (Global Strength S = 16.29 vs. 15.58, *p* = 0.033), where ethical concerns and anxieties were diffusely interconnected. In contrast, experts demonstrated a “low-density, strong-structure” network, characterized by significantly stronger associations along specific “application-ethics” pathways.

**Conclusion:**

The cognitive architecture of AI perception differs fundamentally by expertise level. For public health systems to effectively implement AI, “one-size-fits-all” training is insufficient. Strategies must shift toward stratified cognitive reshaping: providing structured scaffolding to manage diffuse anxiety in novices, and empowering experts to lead evidence-based evaluation and clinical implementation.

## 1 Introduction

Artificial Intelligence (AI) is permeating all levels of healthcare at an unprecedented speed, fundamentally reshaping clinical practice, disease diagnosis, and patient care models (1, 2). In the nursing field, AI technology demonstrates immense potential to enhance nursing quality and efficiency by optimizing clinical workflows, aiding decision support, automating routine tasks, and enabling personalized patient monitoring (1, 3). As a key area for technological application, the integration of AI in maternal and child health is expected to provide more precise and timely health services for pregnant women, mothers, and children. However, the successful integration of AI technology depends not only on the maturity of the technology itself but, more critically, on the acceptance, literacy, and attitudes of frontline nursing staff, the largest group within the healthcare system (4, 5). This study explicitly distinguishes between these two core variables: Artificial Intelligence Literacy serves as a cognitive competency indicator, encompassing an individual’s ability to understand, use, evaluate, and ethically apply AI technologies (6); conversely, Attitudes toward Artificial Intelligence function as a psychological disposition indicator, reflecting nurses’ psychological acceptance or emotional resistance toward this emerging technology (7). This distinction is critical because “knowing AI” does not necessarily equate to “trusting AI.” Integrating both components to construct a “cognitive-attitude” framework is a prerequisite for a comprehensive assessment of the digital readiness of nursing human resources.

Despite AI’s great potential for improving efficiency and patient care, its widespread adoption in healthcare faces significant challenges. This is primarily due to prevalent psychological barriers among healthcare professionals (such as anxiety, fear, and mistrust) and the behavioral resistance that follows (8, 9). A systematic review pointed out that the nursing community generally holds contradictory attitudes toward AI: on one hand, they are positive about AI’s supportive role; on the other hand, they generally lack AI knowledge and hold significant concerns about the technology (10). These concerns involve multi-level barriers, including worries about algorithmic bias, data privacy breaches, ethical responsibilities, and AI explainability (11). Deeper barriers stem from clinicians’ psychological reactions to AI, such as “AI Anxiety” and potential impacts on patient safety (12). Research in nursing education shows that nursing students harbor significant “AI fear” (13). This fear primarily revolves around two core dimensions: first, the threat to their professional roles and future job security, and second, concerns that AI applications will lead to the dehumanization of nursing workflows and the loss of human traits like empathy. This negative cognition, centered on “fear,” may become a decisive factor hindering the clinical implementation of AI technology.

It is particularly noteworthy that regarding technology adoption, the nursing community is not a homogenous group. Based on the renowned “Novice to Expert” theoretical framework of nursing practice, nurses with different experience levels exhibit fundamental differences in their adoption and application of information technology (IT) (14). Specifically, Clinical Preceptors, as experienced experts, have highly internalized clinical knowledge and cognitive patterns; whereas Nurse Interns, as novices, are still in the construction phase of knowledge and skills. Furthermore, Nurse Interns, as members of the younger generation, are often considered “digital natives” (15), which echoes the increasingly prominent theme of generational differences in healthcare. Therefore, when these two groups confront AI, a disruptive technology, their cognitive structures (i.e., how different psychological nodes such as AI literacy, attitudes, and fear interconnect) may fundamentally differ. However, most previous studies (4, 5, 10) have predominantly relied on calculating total scores from traditional instruments, such as literacy scales or attitude scales, and utilized mean comparisons or descriptive statistics to examine the current state of nurses’ AI perceptions. Although these investigations have shed light on the general attitude and knowledge levels regarding AI within the nursing workforce, they are limited by viewing psychological traits as isolated, linear summations. As a result, they overlook the intricate internal relationships and dynamic interactions between various psychological variables, such as technological dread, ethical concerns, and implementation intent. Furthermore, they have yet to uncover how professional experience levels moderate the topological structure and stability of these cognitive networks from a systemic viewpoint.

Therefore, this study aims to fill this gap. We are the first to adopt psychological network analysis (PNA), shifting away from treating AI literacy and attitudes as isolated metrics and instead viewing them as a complex dynamic system. The objectives of this study are: (1) To construct cognitive network models of AI literacy and attitudes for maternal and child health (MCH) nurses (Clinical Preceptors and Nurse Interns); (2) To accurately identify the core nodes (e.g., “fear”) that play a dominant role in these networks; and (3) To quantitatively compare the differences in network structure, core nodes, and connection patterns between Clinical Preceptors and Nurse Interns. By revealing the network structural differences in AI-related cognition (particularly fear) among nurses with varying levels of experience, this study expects to provide empirical evidence for developing targeted AI education and intervention strategies. The goal is to alleviate technology-related anxiety and promote the successful integration of AI in the field of MCH nursing. Furthermore, identifying these cognitive structures not only provides a systemic evidence base for public health administrators to formulate targeted policies for healthcare workforce digital readiness but, more importantly, ensures the precise and efficient application of AI-assisted tools in clinical practice by building a nursing team with high digital preparedness. This digital empowerment of the nursing workforce will translate directly into improved clinical decision-making quality and reduced medical risks, ultimately tangibly enhancing health outcomes for maternal and child patients and realizing the clinical value transformation of smart healthcare technologies.

## 2 Methods

### 2.1 Methodological framework

This large-scale multicenter cross-sectional study was conducted in 32 maternal and child health (MCH) institutions across 26 provinces in China, providing comprehensive geographic coverage of public health nursing personnel. Data collection occurred from January 1 to March 1, 2025, using a convenience sampling method.

### 2.2 Participants

#### 2.2.1 Sample overview

A total of 1,031 eligible participants were ultimately included in this study. The sample consisted of 498 clinical preceptors and 533 nurse interns.

#### 2.2.2 Inclusion and exclusion criteria

The specific inclusion and exclusion criteria for each group are as follows:

1. Clinical Preceptors:

Inclusion criteria: Registered nurses currently employed at MCH care institutions in mainland China; possessing ≥1 year of clinical teaching (preceptorship) experience.

Exclusion criteria: ① cumulative leave exceeding 6 months within the past year; ② currently participating in standardized residency nurse training, departmental rotation, advanced training, currently on probation, or re-employed after retirement; ③ previous participation in AI-related questionnaire surveys on the same topic.

1. Nurse Interns:

Inclusion criteria: Full-time nursing students (associate degree/bachelor’s degree or higher) currently enrolled in mainland China; having been exposed to medical AI-related scenarios through courses, internships, or self-study.

Exclusion criteria: ① diagnosed with mental illnesses such as anxiety or depression within the last 6 months, or cumulative sick leave exceeding 6 months in the last 12 months; ② currently pursuing a dual degree in computer science or biomedical engineering; ③ previous participation in AI-related medical questionnaire surveys on the same topic.

#### 2.2.3 Participant characteristics

Detailed demographic characteristics of the participants in both groups are presented in Supplementary Table S1. The clinical preceptors were predominantly female (98.4%, 490/498), with ages primarily concentrated between 31 and 40 years (59.0%, 294/498). The majority were married (87.1%, 434/498), possessed over 11 years of professional experience (66.2%, 330/498), and primarily held intermediate professional titles (57.8%, 288/498). Regarding their clinical background, the highest proportion of preceptors worked in pediatric departments (49.2%, 245/498), and their workplaces were mainly tertiary Grade A comprehensive hospitals (54.4%, 271/498). Notably, 41.8% (208/498) of the clinical preceptors reported having no prior exposure to artificial intelligence (AI)-related training.

Among the nurse interns, females accounted for 87.6% (467/533), with ages mainly concentrated between 20 and 22 years (45.8%, 244/533). Most interns (83.5%, 445/533) came from two-parent families, and 69.0% (368/533) held rural residency. Additionally, 67.2% (358/533) of the nursing interns had an educational background of an associate degree or below. Their internship rotations were primarily in obstetrics and gynecology (41.8%, 223/533) and pediatrics (49.5%, 264/533), with 67.4% (359/533) interning at tertiary Grade A hospitals. Compared to the clinical preceptors, a lower proportion of nurse interns had never received specific AI training (32.6%, 174/533).

### 2.3 Research instruments

The survey questionnaire used in this study comprised three parts: a General Information Questionnaire, the Artificial Intelligence Literacy Scale (AILS), and the General Attitudes toward Artificial Intelligence Scale (GAAIS).

#### 2.3.1 General information questionnaire

This questionnaire was self-designed by the researchers. For Clinical Preceptors: The collected information included gender, age, ethnicity, marital status, professional title, years of work, working department, hospital nature (e.g., public/private), hospital level, and AI-related training experience. For Nurse Interns: The collected information included gender, age, ethnicity, family type, household registration (hukou) location, current educational level, internship department, internship hospital type, internship hospital level, and AI-related training experience.

#### 2.3.2 Artificial intelligence literacy scale

This study employed the AILS developed by Wang et al. (6) to assess the AI literacy levels of clinical preceptors and nurse interns. The reliability and validity of this scale have been verified in related studies (16, 17). The AILS contains 12 items, divided into four core dimensions, with each dimension consisting of 3 specific items: ① awareness (corresponding to items AW_1–AW_3): measuring an individual’s perception and identification of AI’s existence; ② usage (corresponding to items US_1–US_3): measuring an individual’s ability to operate and apply AI tools; ③ evaluation (corresponding to items EV_1–EV_3): measuring an individual’s capacity for critical assessment of AI outputs; ④ ethics (corresponding to items ET_1–ET_3): measuring adherence to privacy, security, and ethical standards during AI application. This scale moves beyond foundational knowledge by placing significant emphasis on the “Ethics” and “Evaluation” dimensions. Such a focus is particularly critical for maternal and child health (MCH) nurses, who frequently navigate high-risk clinical scenarios and high-stakes decision-making environments. The scale utilizes a 7-point Likert scoring method (from 1 “Strongly disagree” to 7 “Strongly agree”). Items AW_2, US_2, and ET_2 are reverse items and require reverse-coding during scoring. The total score for the scale ranges from 12 to 84, with higher scores indicating higher levels of AI literacy. The original scale’s Cronbach’s *α* value was 0.830, showing good internal consistency.

#### 2.3.3 General attitudes toward artificial intelligence scale

This study employed the GAAIS, developed by Schepman and Rodway (7), to assess the overall attitudes toward AI among MCH clinical preceptors and nurse interns. This scale has been confirmed to effectively measure both positive and negative attitudes of nurses toward AI (17). The GAAIS consists of a total of 20 items, divided into two sub-dimensions: ① positive attitude (corresponding to items P_1–P_12, 12 items): reflecting an individual’s optimistic expectations regarding the benefits, convenience, and future development brought by AI technology. ② negative attitude (corresponding to items N_1–N_8, 8 items): primarily used to identify “AI fear,” technical anxiety, and deep-seated concerns about ethical risks. The bidirectional design of the scale, encompassing both positive and negative dimensions, allows for the precise capture of pervasive, deep-seated anxieties such as “AI fear” among nurses, a feat that is difficult to achieve with traditional unidimensional attitude scales. The scale uses a 5-point Likert scoring method (from 1 “Strongly disagree” to 5 “Strongly agree”). During scoring, all items in the negative attitude (N_1–N_8) sub-dimension must be reverse-coded. The total score for the scale ranges from 20 to 100, with higher scores indicating more positive attitudes toward AI. In the original scale, the Cronbach’s *α* value was 0.880 for the positive attitude dimension and 0.830 for the negative attitude dimension.

### 2.4 Translation and pilot study of the scales

#### 2.4.1 Translation and validity evaluation

In this study, the AILS and the GAAIS were translated, back-translated, and culturally adapted in accordance with the World Health Organization (WHO) guidelines for the cross-cultural adaptation of research instruments. Subsequently, six experts with extensive experience and senior professional titles in fields such as artificial intelligence, psychology, and sociology were invited to evaluate the content validity of the scales. The results indicated that for the AILS, the Item-level Content Validity Index (I-CVI) ranged from 0.83 to 1.00, and the Scale-level Content Validity Index (S-CVI) was 0.983. For the GAAIS, the I-CVI ranged from 0.83 to 1.00, and the S-CVI was 0.986. These results demonstrate that both scales possess excellent content validity.

#### 2.4.2 Pilot study implementation and reliability testing

Following the principle that the sample size for a pilot study should constitute 10 to 30% of the planned formal investigation (18, 19), this study recruited 50 clinical preceptors and 50 nurse interns from a tertiary-grade maternal and child health institution in Chengdu for pilot testing. Based on the feedback obtained, necessary linguistic refinements were made to the questionnaire to ensure that all items were accurately phrased and easily understood.

The results of the pilot study indicated that the Cronbach’s *α* coefficient for the overall AILS was 0.827 (0.825 in the formal investigation). For the GAAIS, the overall Cronbach’s α was 0.828 (0.882 in the formal investigation), with α coefficients of 0.957 for the positive attitude dimension and 0.961 for the negative attitude dimension. These findings demonstrate that the Chinese versions of the scales possess high internal consistency reliability among the target population.

### 2.5 Data collection and quality control

#### 2.5.1 Sample size calculation

$n={\left[\frac{Z_{1-\alpha /2}}{\delta }\right]}^{2}\times p\times (1-p)$

The sample size was calculated based on the standard formula for cross-sectional studies (20, 21). The parameters were set as follows: Z_1-*α*/2_ = 1.96 (corresponding to a 95% confidence level), *p* = 0.5 (maximum variation rate), and *δ* = 0.05 (5% absolute margin of error). The theoretical minimum sample size was calculated to be 385. Considering a potential 20% rate of invalid questionnaires or participant attrition common in cross-sectional surveys, the final target sample size was determined to be at least 462. This study actually included 1,031 participants, providing a sufficient sample size that meets the necessary statistical requirements.

#### 2.5.2 Implementation of the formal investigation

A multicenter cross-sectional survey was conducted from January 1 to March 1, 2025, using the “Wenjuanxing” online platform^1^ across 32 maternal and child health (MCH) institutions in 26 provinces in China. Once the questionnaire was generated as a QR code, it was distributed via email and WeChat to the nursing directors of each participating institution, who then shared it within their respective departments to invite eligible clinical preceptors and nursing interns to participate voluntarily. The first page of the questionnaire featured an electronic informed consent form, providing detailed information regarding the research objectives and privacy protection measures; respondents could only proceed to the survey after clicking “I Agree.” To ensure data completeness, a paging design was utilized, and all items were set as mandatory. Preliminary testing indicated that the average time for participants to complete the questionnaire (comprising 32 scale items and general information) was approximately 6–10 min. To fully ensure a positive participant experience and accommodate those with slower reading speeds or those requiring additional time for reflection, the Wenjuanxing platform was configured in “no time limit” mode. This ensured that the platform would not automatically force an exit or time out due to an extended duration; the session remained active until the participant proactively clicked “Submit.”

#### 2.5.3 Quality control and data cleaning

To ensure the reliability of the research findings, a rigorous logical cleaning process was performed after data exportation based on the following criteria:

1. Technical Control: The “limit to one response per IP address” function on the Wenjuanxing platform was enabled to prevent duplicate submissions.
2. Questionnaires with abnormal completion durations were excluded based on the following criteria: ① durations under 3 min (*n* = 45): This was deemed below the minimum threshold for adequate reading and response as determined in the pre-test. Such instances were considered indicative of careless or random completion, which could not guarantee data quality. ② durations exceeding 30 min: The 30-min upper limit was established primarily to minimize external interference. Given that online surveys are frequently completed during fragmented intervals, an excessively long duration (more than triple the average time) typically signifies significant interruptions, such as addressing clinical emergencies or answering phone calls. Such disruptions can result in a lack of psychological continuity or increased susceptibility to environmental factors, thereby introducing potential measurement bias.
3. Response Pattern and Logic Checks: Questionnaires exhibiting obvious patterns (e.g., selecting the same option for all items) or internal logical contradictions were removed.
4. Eligibility Screening: Samples failing to meet the inclusion/exclusion criteria were excluded. This included 5 clinical preceptors without teaching experience and 10 who were in standardized training, rotational shifts, advanced studies, probationary periods, or re-employment after retirement. Additionally, 39 nurse interns who had not yet participated in actual clinical rotations were excluded.

Ultimately, a total of 1,130 questionnaires were collected. After excluding 99 invalid responses, 1,031 valid questionnaires remained (including 498 from clinical preceptors and 533 from nurse interns), resulting in an effective recovery rate of 91.2%.

#### 2.5.4 Definitions of theoretical frameworks and analytical models

To ensure the interpretability and logical consistency of the research findings, the following theoretical models and analytical methods were incorporated into this study:

1. Scaffolding Theory: Proposed by Wood et al. (22), this theory refers to the process in which temporary support (scaffolding) is provided to help learners bridge their “Zone of Proximal Development” and eventually achieve independent mastery while acquiring new knowledge. In this study, Scaffolding Theory serves as the theoretical basis for explaining how novices (nurse interns) can alleviate pervasive AI-related anxiety and progressively build AI literacy through structured educational interventions.
2. Train-the-Trainer (TTT) Model: The TTT model is a multiplier educational framework designed to train core experts (trainers), enabling them to effectively transfer knowledge to other learners (23). In this study, clinical preceptors are defined as “experts” based on the logic of the TTT model, exploring the feasibility of utilizing them as “seed teachers” to lead the AI transformation of nursing teams and facilitate the translation of knowledge into clinical practice.
3. Independent Groups Gaussian Network Comparison Test (IG-NCT): IG-NCT is a psychological network comparison technique based on permutation testing (24). It allows researchers to statistically evaluate whether significant differences exist between two independent groups (e.g., the experts and novices in this study) in terms of Network Topology and Global Strength. This approach transcends traditional mean comparisons, revealing the fundamental distinctions in the AI cognitive architecture across nurses with different experience levels from a system-level perspective.

### 2.6 Statistical analysis

This study used SPSS 27 and R software (version 4.4.3) for data analysis. All tests were two-tailed, and a *P*<0.05 was considered statistically significant. Network analysis was primarily implemented using the qgraph and bootnet packages in R. First, descriptive statistics were performed on all variables (continuous variables were expressed as mean ± standard deviation, and categorical variables as frequencies and percentages). To assess the degree of variation for each scale item, the standard deviation was used as an indicator of information content. Inter-item correlations were also examined to ensure no redundant item (25).

#### 2.6.1 Estimation of AILS and GAAIS network structures

Network analysis is a data-driven approach adept at revealing the independent relationships and interactions among multiple variables within a complex system (26). This study utilized network analysis to explore the association patterns among the 32 items of the AILS and GAAIS, and how they collectively influence the AI literacy and attitude levels of clinical preceptors and nurse interns. The analysis was conducted using the qgraph package in R to construct and visualize the network structures. A LASSO (Least Absolute Shrinkage and Selection Operator) regularized partial correlation analysis was employed to build the AILS and GAAIS networks. This regularization technique reduces noise and generates a sparser (more interpretable) network, thereby highlighting key item associations (26). Model selection was optimized using the Extended Bayesian Information Criterion (EBIC) to ensure the model maintained parsimony while fitting the data (25). Network visualization was based on the Fruchterman-Reingold algorithm (a “spring” layout), which positions nodes (items) with greater influence at the center of the network and places strongly associated nodes closer to each other. In the network graphs: Nodes: Represent the 32 items from the AILS and GAAIS. Edges (Lines): Represent the partial correlation coefficients between two items after controlling for the influence of all other items. Edge thickness: Reflects the strength of the association (thicker lines for stronger associations, thinner lines for weaker ones). Edge color: Green edges indicate positive correlations, while red edges indicate negative correlations.

#### 2.6.2 Characterization and validation of AILS and GAAIS network structures

This study characterized the network properties using three core indices:

Strength: The sum of the absolute weights of all edges connected to a node, which reflects the overall influence of that item in the network (27).

Bridge Strength: The sum of the edge weights connecting a node to nodes in other dimensions (communities), which measures the item’s key role in cross-dimensional influence (28).

Predictability: Reflects the extent to which an item can be predicted by other items in the network (29).

To ensure the reliability and stability of the network analysis results, this study adopted edge accuracy assessment and centrality stability analysis (25, 30). Edge accuracy was assessed using 95% non-parametric confidence intervals (CIs) based on 1,000 bootstrap samples; narrower CIs indicate more precise edge weight estimations. Centrality stability was examined using the correlation stability coefficient (CS-coefficient) to test node strength. A CS-coefficient higher than 0.25 indicates acceptable results, while one higher than 0.5 is considered ideal. For comparisons of edge weights and node strength, difference tests were conducted using bootstrap CIs. If the CI did not contain zero, the difference was considered statistically significant.

#### 2.6.3 Network comparison analysis between clinical preceptors and nurse interns

To investigate the differences in the AI literacy and attitude network structures between Clinical Preceptors and Nurse Interns, this study utilized the NetworkComparisonTest package in R to conduct an independent-group Gaussian NET. This method was used to generate the respective network structure graphs for both groups and to perform a difference analysis on centrality indices, such as node strength (Strength) and bridge strength (Bridge Strength).

## 3 Results

### 3.1 Descriptive statistics and preliminary analysis of scales

The descriptive statistics for the 32 items of the AILS and GAAIS, including means, standard deviations, and t-test comparisons between the two groups, are detailed in Supplementary Table S2. Preliminary analysis confirmed the data quality: the standard deviations of all items were within a reasonable range, and no items with excessively low information content (i.e., standard deviation 2.5 SD below the mean) were found. The inter-item correlation coefficients were all less than 0.25, indicating no redundant items were detected, and suggesting that each item independently measures different dimensions. Regarding the total scores for AI literacy and attitudes:

1. AI Literacy (AILS): The total score for Clinical Preceptors was significantly higher than that of Nurse Interns (56.10 ± 9.50 vs. 54.94 ± 8.23, *t* = 2.100, *p* = 0.036). Preceptors performed particularly better in the Ethics dimension (ET_1: 5.48 ± 1.17 vs. 5.09 ± 1.21, *t* = 5.151, *p* < 0.001) and in the ability to evaluate AI technology applications (EV_1–3, all *p* < 0.05).
2. AI Attitudes (GAAIS): Clinical Preceptors had a significantly higher total score for positive attitudes (70.51 ± 10.62 vs. 66.61 ± 8.85, *t* = 6.382, *p* < 0.001).
3. Specific Differences: Nurse Interns perceived AI technology as less difficult to learn (US_2: 3.88 ± 1.30 vs. 3.65 ± 1.49, *t* = −2.582, *p* = 0.010). Significant differences (*p* < 0.01) were observed between the two groups in the negative attitude dimensions (e.g., N_3–N_8).

### 3.2 Overall network characteristics of AILS and GAAIS

#### 3.2.1 Network structure features

**Figure 1:**
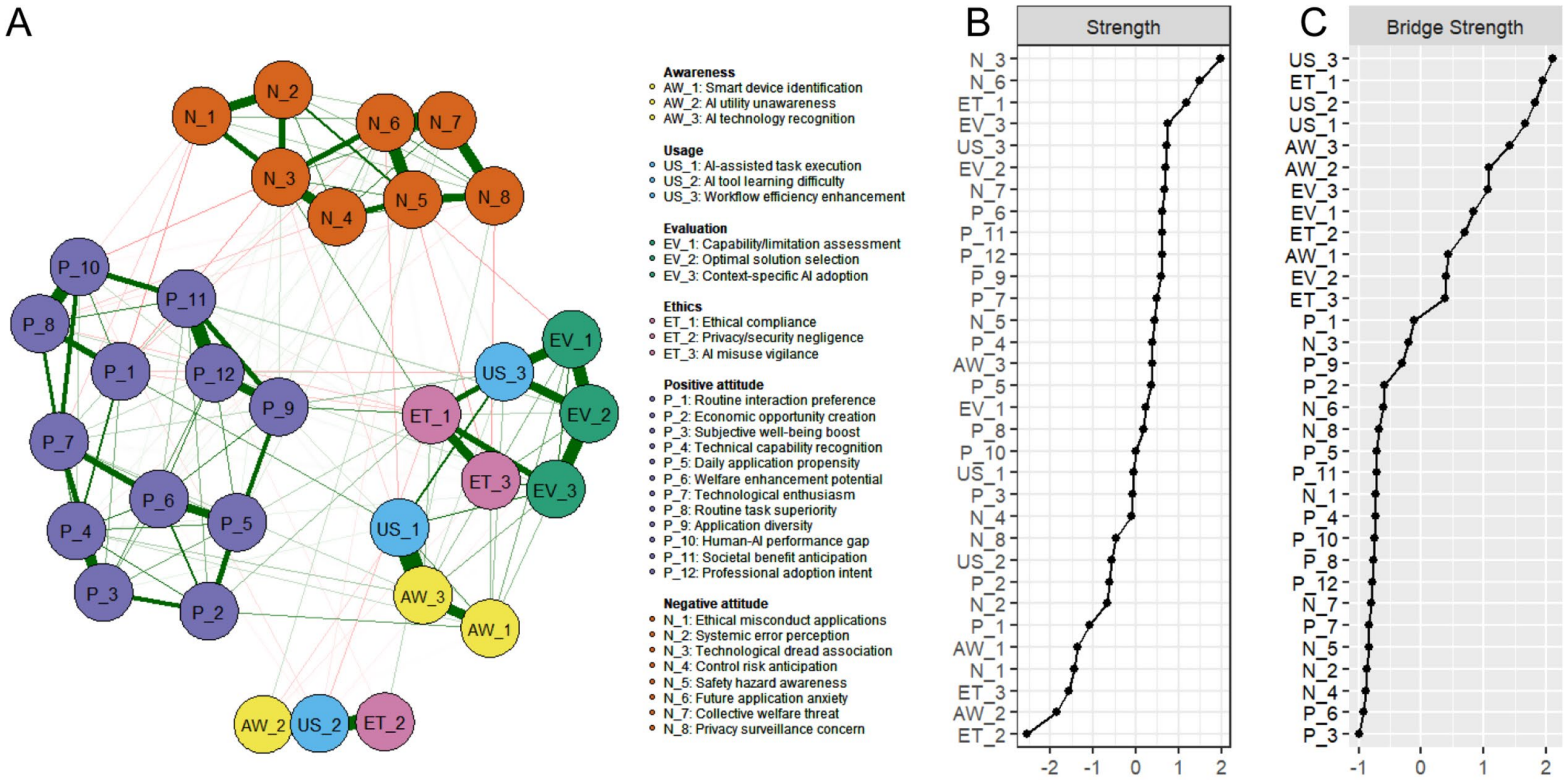
The AILS and GAAIS network diagram (*N* = 1,031). Only edges with weights ≥ 0.01 are displayed. **(A)** The network structure comprising 32 nodes, constructed using the qgraph package based on a partial correlation matrix. **(B)** Strength centrality for the 32 nodes in the network. **(C)** Bridge strength centrality for the 32 nodes in the network.

Figure 1A displays the network structure of the AILS and GAAIS based on the total sample (*N* = 1,031). The network comprises 32 nodes, representing the 32 items for AI literacy and attitudes, covering the four dimensions from AILS—Awareness (AW_1 – AW_3), Usage (US_1 – US_3), Evaluation (EV_1 – EV_3), and Ethics (ET_1 – ET_3), and the two dimensions from GAAIS—Positive attitude (P_1 – P_12) and Negative attitude (N_1 – N_8). There are a total of 179 non-zero edges in the network (accounting for 36.1% of all possible edges, i.e., 179/496), with an average edge weight of 0.029. This indicates a moderate degree of systematic association among the different AI literacy and attitude items. The network exhibits a pattern of “sparse strong connections + dense weak connections.” In the graph, green edges represent positive correlations and red edges represent negative correlations. The deeper the color and the thicker the width of an edge, the higher the strength of the association.

#### 3.2.2 Strength centrality analysis

Strength centrality (Strength) reflects the overall influence of a node within the network. Figure 1B and Supplementary Table S3 present the Strength centrality for the 32 nodes. The results show that N_3 (Technological dread association), from the “Negative attitude” dimension of the GAAIS, possesses the highest Strength centrality (Strength = 1.978). This indicates that it plays a core role in the AILS and GAAIS network and has the greatest impact on the overall cognitive attitude. This was followed by N_6 (Future application anxiety; Strength = 1.484) and ET_1 (Ethical compliance) from the “Ethics” dimension of the AILS (Strength = 1.197).

#### 3.2.3 Bridge strength analysis

Bridge Strength measures a node’s ability to connect different dimensions (communities) and is a key indicator of cross-dimensional influence. Figure 1C and Supplementary Table S3 display the analysis results for Bridge Strength. US_3 (Workflow efficiency enhancement), from the “Usage” dimension of the AILS, exhibited the highest Bridge Strength (Bridge Strength = 2.102). This suggests it plays the most critical bridging role in connecting AI technology application with AI attitudes. This was followed by ET_1 (Ethical compliance) from the “Ethics” dimension (Bridge Strength = 1.948) and US_2 (AI tool learning difficulty) from the “Usage” dimension (Bridge Strength = 1.821).

#### 3.2.4 Node predictability

**Figure 2:**
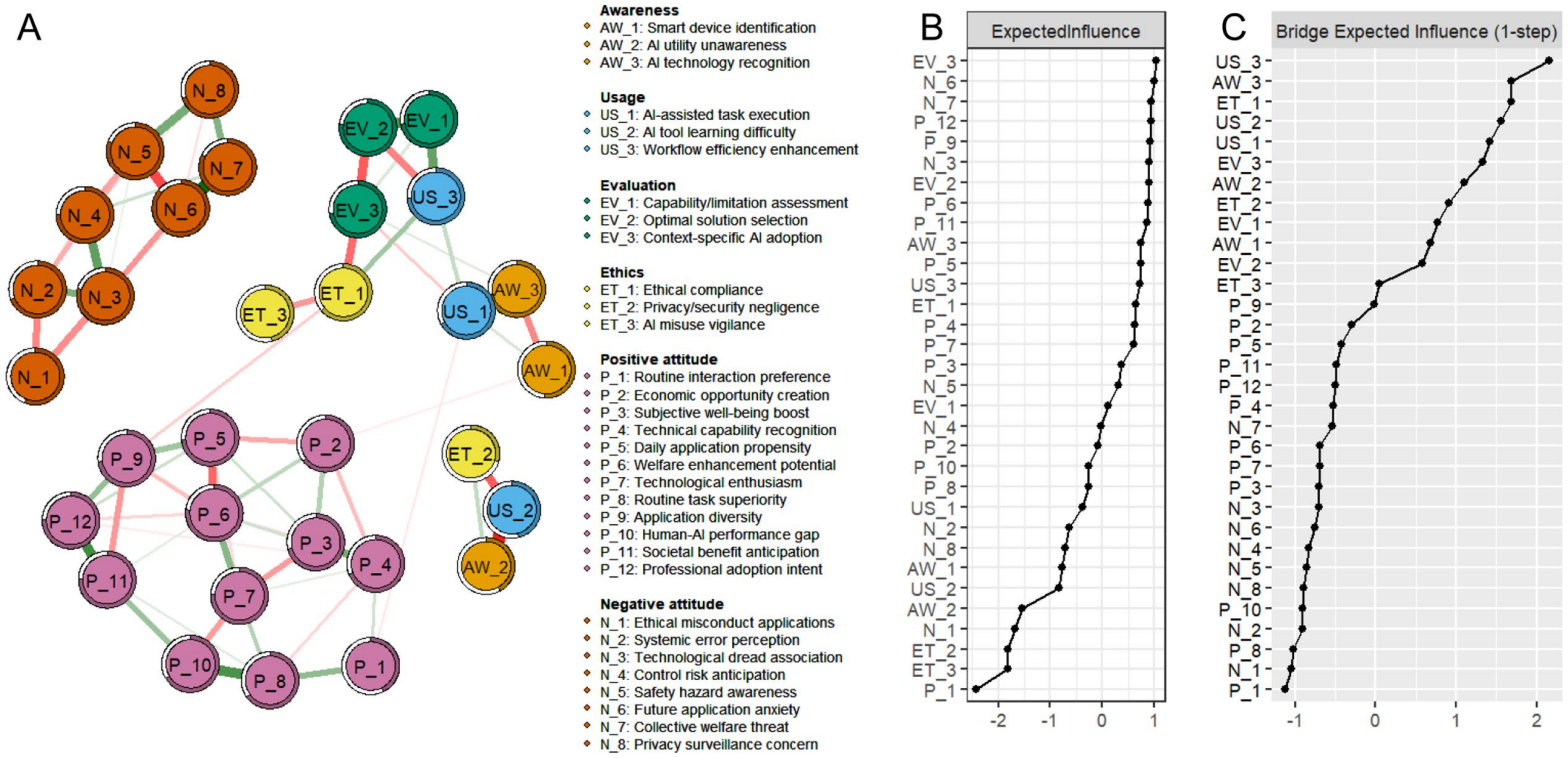
Network structure with node predictability and influence estimates (*N* = 1,031). Only edges with weights ≥ 0.01 are displayed. **(A)** Network structure with the 32 nodes interactions and attributes. **(B)** Expected influence of each node. **(C)** Bridge expected influence (1-step).

Node predictability (Predictability) reflects the extent to which a node can be explained by the other nodes in the network. Figure 2A and Supplementary Table S3 show the predictability of the 32 nodes, represented by the circles around the nodes, while the corresponding node influence estimates are presented in Figures 2B,C. The predictability values for all nodes ranged from 29.3 to 86.1%. N_6 (Future application anxiety), from the “Negative attitude” dimension of the GAAIS, had the highest predictability (R^2^ = 0.861), meaning that 86.1% of its variance can be explained by the other items in the network. This was followed by N_7 (Collective welfare threat, R^2^ = 0.821) and N_5 (Safety hazard awareness, R^2^ = 0.809). This indicates that individuals’ fear and anxiety regarding AI are highly correlated with the other cognitive and literacy items in the network.

### 3.3 Difference analysis of network characteristics based on experience level

**Figure 3:**
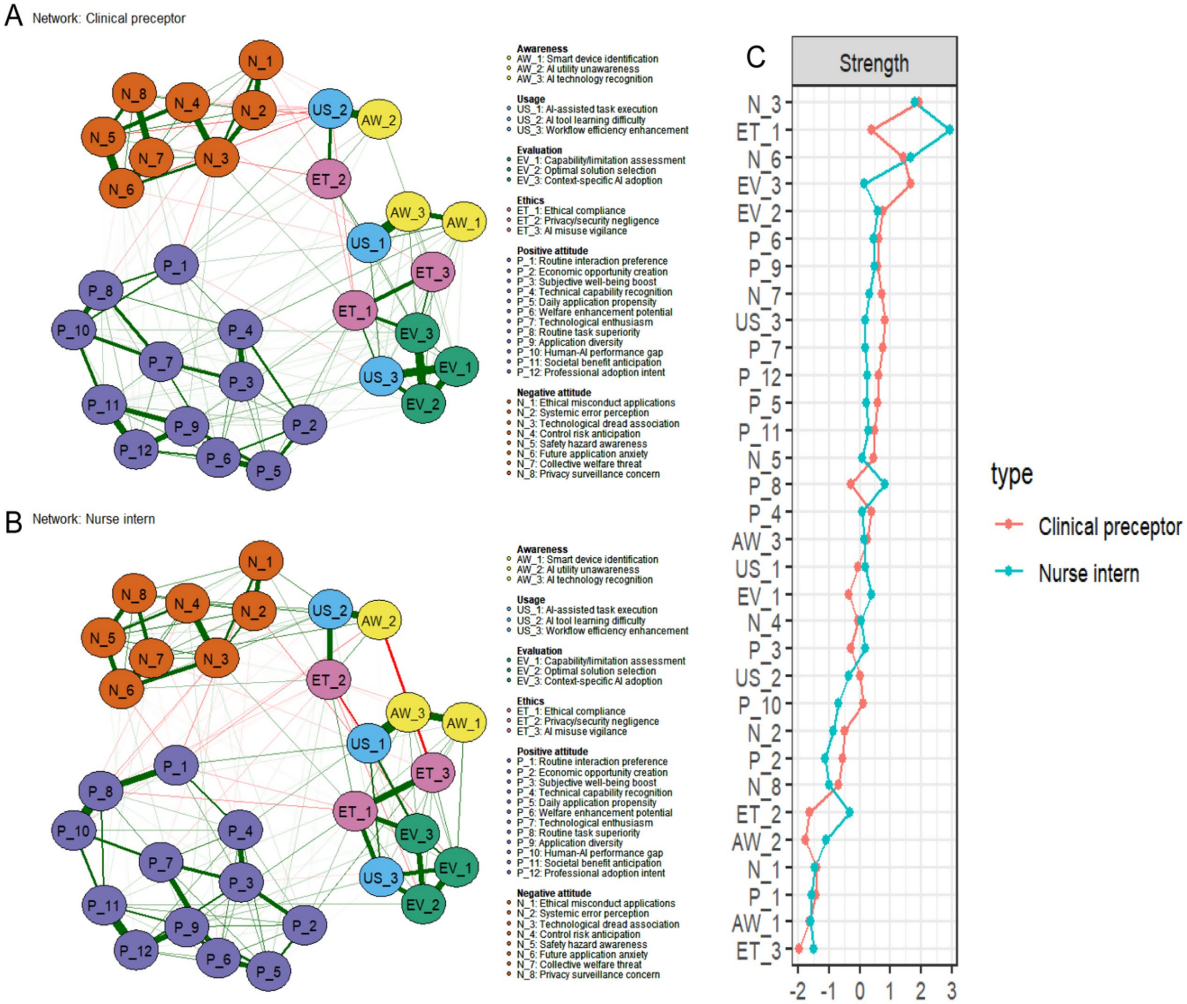
Comparison of network structure and influence by identity level. Only edges with weights ≥ 0.01 are displayed. **(A)** Network for clinical preceptor (*N* = 498), **(B)** Network for nurse intern (*N* = 533), **(C)** Strength by identity level.

The Independent Groups Gaussian Network Comparison Test (IG-NCT) was employed to analyze the network differences between the Clinical Preceptor group (Figure 3A) and the Nurse Intern group (Figure 3B).

Overall Network Structure: The two groups showed significant differences in their overall network topology (M = 0.279, *p* = 0.023), indicating that the association patterns among variables are group-specific.

Global Connection Strength: The Nurse Intern group exhibited stronger overall global connectivity than the Clinical Preceptor group (15.58 vs. 16.29, S = 0.710, *p* = 0.033). This suggests a tighter co-activation among AI-related cognitive and attitude variables in nurse interns.

#### 3.3.1 Edge invariance test

Supplementary Table S4 shows that a total of 25 edge weights differed significantly between the two groups (*p* < 0.05). The network analysis revealed that the Clinical Preceptor group demonstrated a systematic cognitive advantage: all edge weights that showed inter-group differences (25 edges) indicated that the association strength between variables was significantly higher in the preceptor group (E > 0). The three most significant differential edges, ranked in descending order by effect size (E), were:

1. US_1 (“AI-assisted task execution”) — ET_2 (“Privacy/security negligence”; E = 0.279, *p* = 0.001).
2. AW_2 (“AI utility unawareness”) — ET_3 (“AI misuse vigilance”; E = 0.189, *p* = 0.001).
3. EV_3 (“Context-specific AI adoption”) — ET_2 (“Privacy/security negligence”; E = 0.161, *p* = 0.001).

#### 3.3.2 Node strength centrality invariance test

Figure 3C illustrates the differences in node strength (Strength) between the two groups. The results show that 5 nodes exhibited significant differences in network strength between the two groups (*p* < 0.05).

1. In the ethical cognition dimension: The strength of ET_1 (“Ethical compliance”; C = −0.403, *p* = 0.001) and ET_3 (“AI misuse vigilance”; C = −0.252, *p* = 0.005) was significantly lower in the preceptor group compared to the intern group.
2. In the technology application dimension: The strength of EV_3 (“Context-specific AI adoption”; C = 0.171, *p* = 0.041) was significantly higher in the preceptor group than in the intern group.
3. In the cognitive regulation dimension: The strength of AW_2 (“AI utility unawareness”; C = −0.208, *p* = 0.044) and P_8 (“Routine task superiority”; C = −0.227, *p* = 0.026) was significantly lower in the preceptor group compared to the intern group.

#### 3.3.3 Bridge strength difference analysis

**Figure 4:**
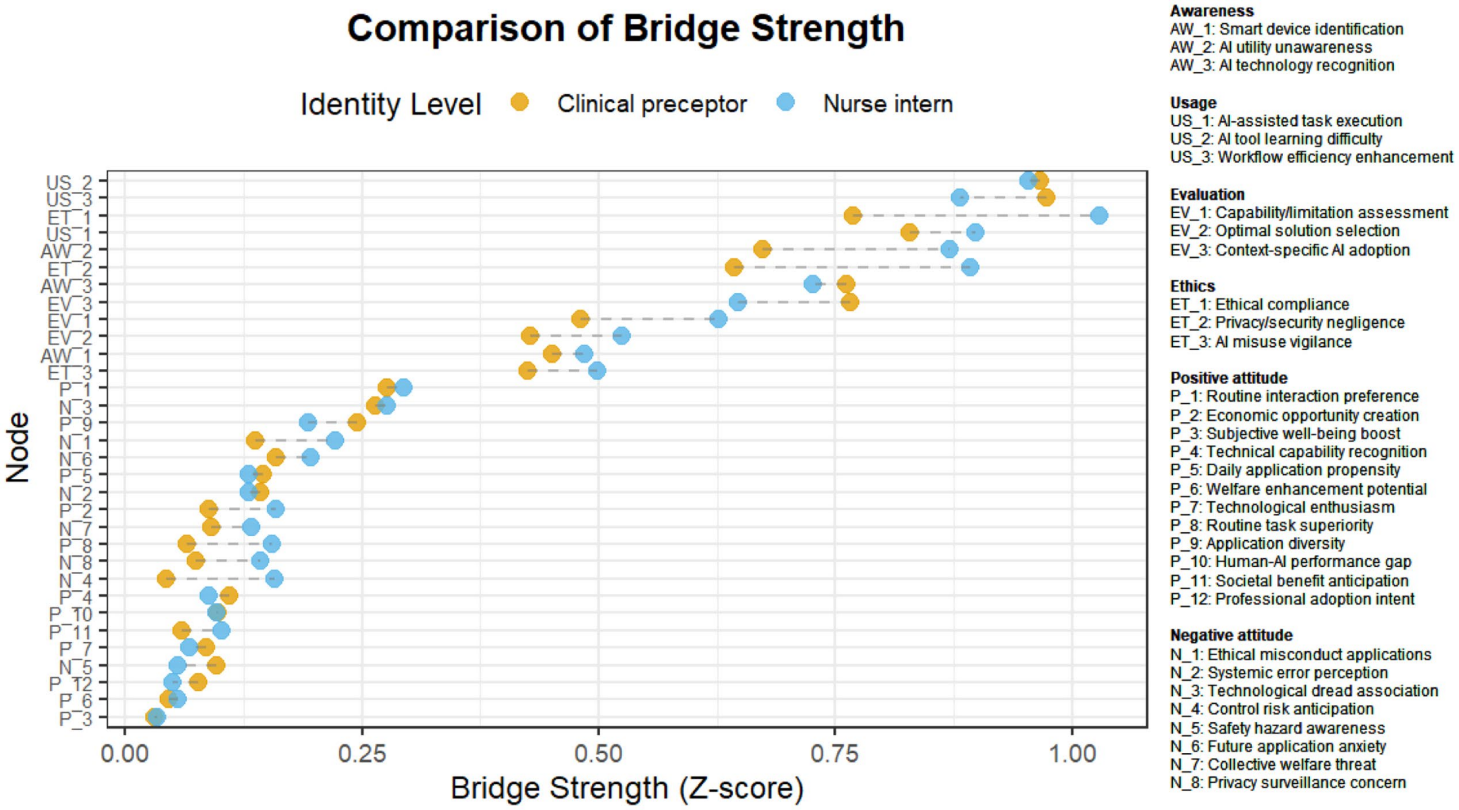
Comparison of bridge strength by identity level.

Figure 4 shows the comparison of bridge strength between the two groups. Overall, there was a significant difference in the total bridge strength for AI literacy and attitudes between the Clinical Preceptor group and the Nurse Intern group (*t* = −2.587, df = 31, *p* = 0.015; MD = −0.040, 95%CI: −0.071 – −0.008; Cohen’s d = −0.125).

Node centrality analysis revealed that the Nurse Intern group had significantly higher bridge centrality in the following 3 nodes compared to the Clinical Preceptor group: ET_1 (Ethical compliance; 0.768 vs. 1.029, *Δ* = −0.261) and ET_2 (Privacy/security negligence; 0.643 vs. 0.892, Δ = −0.249) from the “Ethics” dimension. AW_2 (AI utility unawareness; 0.673 vs. 0.871, Δ = −0.198) from the “Awareness” dimension.

### 3.4 Accuracy and stability analysis of the AILS and GAAIS networks

#### 3.4.1 Edge weight accuracy

**Figure 5:**
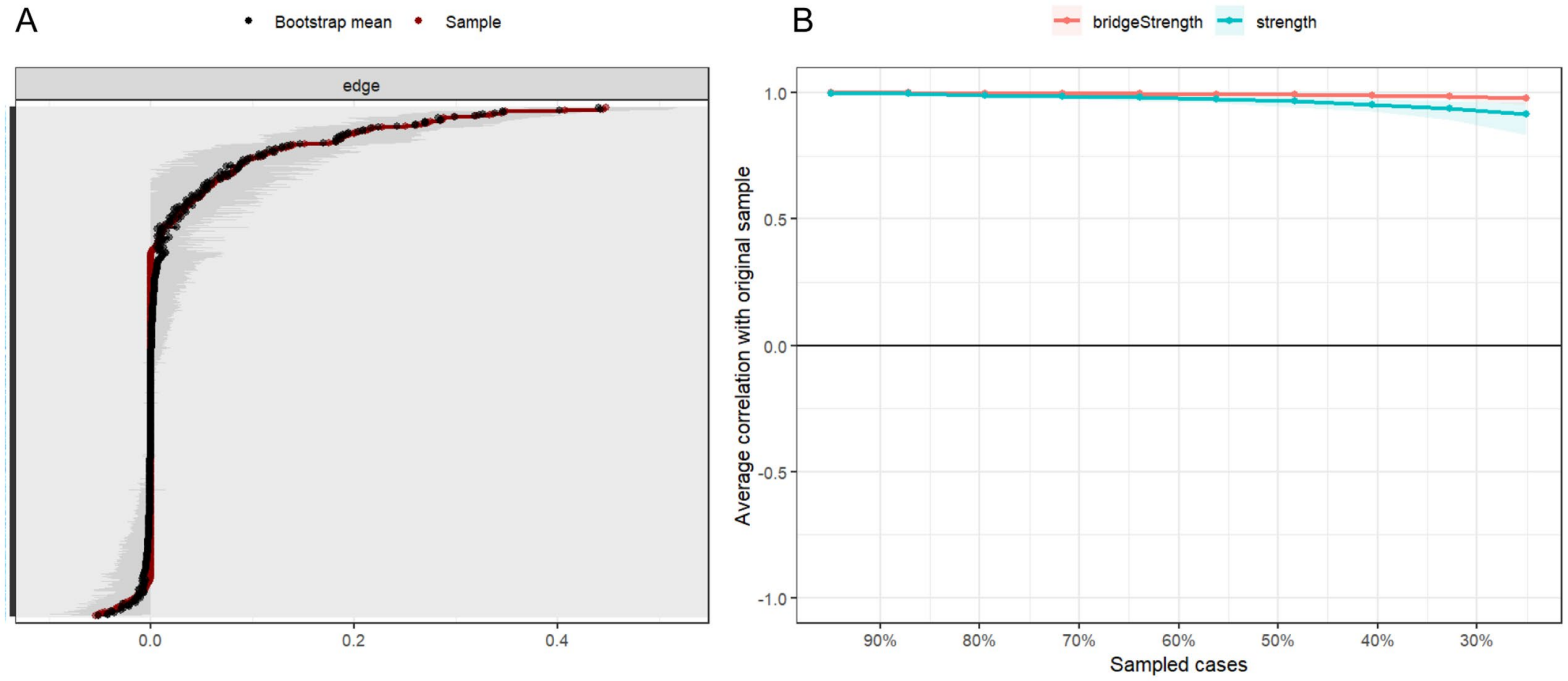
Accuracy and stability analysis of the AILS and GAAIS network (*N* = 1,031). **(A)** Accuracy analysis of the edge weights. **(B)** Stability analysis of the centrality indices.

Figure 5A displays the results of the edge weight accuracy analysis for the network model. The overall connectivity strength of the network was moderate to weak, with a median edge weight of 0.0286 (95% CI: 0.0069–0.0526). The analysis indicated that most edge connections (especially the core association structures) were statistically significant and had narrow CIs, demonstrating high precision and stability in the estimations.

#### 3.4.2 Centrality stability

Figure 5B presents the stability analysis for Strength centrality (Strength) and Bridge Strength. The results showed that the CS-coefficient for Strength centrality was 0.750, and the CS-coefficient for Bridge Strength was also 0.750. Both coefficients far exceed the preferred threshold of 0.5 (25). This indicates that the centrality indices remain highly stable even when the sample size is reduced, confirming the robustness of the network analysis results.

### 3.5 Intra-network difference test (bootstrap difference test)

To further validate the differences within the network structure, this study used a Bootstrap difference test to compare edge weights, node strength, and bridge strength.

#### 3.5.1 Edge weight difference test

Supplementary Figure S1 presents the results of the bootstrap difference test for edge weights. In the matrix plot, black squares indicate a significant difference between the items being compared, while gray squares indicate non-significance. The results showed that some key edge connections in the network exhibited significant characteristics. For example: the edge weight between AW_3 (AI technology recognition; from the “Awareness” dimension) and US_1 (AI-assisted task execution; from the “Usage” dimension) was the highest (0.443, 95% CI: 0.367–0.517), indicating a very strong association between these two items. The connection strength between AW_2 (AI utility unawareness) and US_2 (AI tool learning difficulty) was also high (0.441, 95% CI: 0.375–0.506); and N_6 (Future application anxiety) and N_7 (Collective welfare threat) from the “Negative attitude” dimension also showed a moderately strong and stable association (0.400, 95% CI: 0.322–0.472).

#### 3.5.2 Node strength centrality difference test

Supplementary Figure S2 displays the results of the bootstrap difference test for node strength centrality (Strength). The diagonal of the matrix shows the strength value for each node. The results further verify the central roles of N_3 (Technological dread association; Strength centrality = 1.978; Figure 1B) and N_6 (Future application anxiety; Strength centrality = 1.484; Figure 1B) from the “Negative attitude” dimension. They demonstrated significant differences compared to most other nodes (indicated by the large number of black squares in their corresponding rows), confirming their significant and unique influence within the network.

#### 3.5.3 Node bridge strength centrality difference test

Supplementary Figure S3 presents the results of the bootstrap difference test for node bridge strength. The results showed that US_3 (Workflow efficiency enhancement; Bridge Strength = 2.102; Figure 1C) from the “Usage” dimension and ET_1 (Ethical compliance; Bridge Strength = 1.948; Figure 1C) from the “Ethics” dimension showed significant differences compared to most other nodes (indicated by the predominance of black squares in their corresponding rows). This indicates that these two nodes play a statistically significant bridging role in connecting different dimensions.

## 4 Discussion

This study, for the first time, utilizes PNA to deeply analyze and compare the cognitive network structures of Artificial Intelligence Literacy (AILS) and Attitudes toward AI (GAAIS) between clinical preceptors (experts) and nurse interns (novices) in maternal and child specialty nursing. The findings not only quantify the complex relationships among AI-related psychological variables but also reveal cognitive structural differences dominated by experience level. The core findings of this study are twofold:

1. The core position of “AI Fear”: Among all maternal and child nurses, “AI Fear” (specifically N_3 “Technological dread association” and N_6 “Future application anxiety”) was the core node with the strongest influence in the entire cognitive network, dominating nurses’ other literacy and attitudes toward AI.
2. The “Novice-Expert” cognitive differentiation: The network of nurse interns (novices) exhibited a “high-density, undifferentiated” characteristic (i.e., higher global connection strength); whereas the network of clinical preceptors (experts) presented a “low-density, strong-structure” pattern, meaning weaker global connectivity but significantly stronger specific connections in key cognitive pathways (such as “application-ethics” associations).

### 4.1 Finding 1: AI fear—the dominant core of the maternal-child nurse cognitive network

The most critical finding of this study is the quantitative confirmation of the absolute core position of “AI Fear” within the nurses’ AI cognitive system. Strength centrality analysis showed that the node strengths of N_3 (Technological dread association) and N_6 (Future application anxiety) ranked highest among the 32 items. This result provides strong network-structural evidence for the psychological barriers mentioned in the “Introduction”: nurses’ fear and anxiety toward AI are not isolated emotional reactions but rather the “Organizing Hub” of the entire AI cognitive system. This quantitative finding validates the conclusions of previous research, namely that “AI fear” (13) and “AI anxiety” (12) are decisive psychological factors hindering the promotion of AI technology in the medical field. This study’s network model further reveals that all of the nurses’ perceptions regarding AI literacy (such as usage, evaluation) and positive attitudes are closely linked to this deep-seated sense of fear.

Furthermore, this study, through bridge strength analysis, identified the key pathways connecting the two independent “communities” of “AI Literacy (skills)” and “AI Attitude (emotion).” The results showed that US_3 (Workflow efficiency enhancement) and ET_1 (Ethical compliance) were the two strongest bridge nodes. This profoundly reveals the internal mechanism of the “contradictory attitudes” nurses hold toward AI, as mentioned in the “Introduction” (10): nurses’ acceptance (positive attitude) relies on two core pathways. First, they must perceive clear benefits to their workflow from AI in practice (US_3 Workflow efficiency enhancement), which echoes AI’s potential to optimize clinical workflow (3). Second, they must be convinced that the application of AI aligns with ethical norms and patient safety principles (ET_1 Ethical compliance), which directly corresponds to the widespread concerns in the literature regarding ethical responsibility and data security (2, 11).

### 4.2 Finding 2: the “high-density, undifferentiated” network of novices (nurse interns)

The most important theoretical contribution of this study is the extension of the renowned “Novice to Expert” nursing practice theoretical framework (14) into the domain of AI cognition. The study found that the network of nurse interns (novices) exhibited significantly higher global connection strength. This indicates that when novices face this emerging AI technology, their cognitive structure exists in a “high-density, undifferentiated” state. From the perspective of Cognitive Load Theory, this high connectivity implies a high extraneous cognitive load for novices. In their cognitive system, all variables (literacy, positive attitudes, negative attitudes) are highly interconnected and synergistic, presenting a diffuse characteristic where “a single pull affects the entire system.” This high connectivity means that any single cognition a novice holds about AI (for example, one piece of negative news) could rapidly and broadly influence their overall perception of AI, leading to cognitive system instability. Because their fear is diffusely connected to all other nodes, any AI interaction triggers a systemic emotional response, potentially overwhelming their working memory and hindering effective learning.

### 4.3 Finding 3: the “strong-structure, low-density” network of experts (clinical preceptors)

In contrast to novices, the cognitive network of clinical preceptors (experts), while having lower global connection strength, was significantly stronger in specific key pathways (i.e., “edge weights”) than that of nurse interns. For example, the preceptors’ group had significantly stronger connection strengths on the two “application-ethics” pathways: “US_1 (AI-assisted task execution)—ET_2 (Privacy/security negligence)” and “EV_3 (Context-specific AI adoption)—ET_2 (Privacy/security negligence)” (*p* < 0.01; see Supplementary Table S4). This perfectly corroborates the “Novice to Expert” theory (14): experts’ clinical cognition is highly internalized, forming a differentiated and structured cognitive model. It suggests that experts have developed sophisticated cognitive schemas (or “illness scripts” equivalent for technology). Their sparse but strong connections allow them to efficiently segregate ethical risks from operational tasks, demonstrating adaptive expertise. Their AI cognitive system is more resilient and modular. For experts, AI is not an abstract, generalized threat, but a concrete clinical tool. Their concerns are no longer diffuse fears, but rather are focused on the specific risks (e.g., ET_2 Privacy/security negligence) triggered when applying AI in specific contexts (EV_3 Context-specific AI adoption). This cognitive model enables them to more accurately assess the pros and cons of AI in specific clinical practices, which aligns with the emphasis in the literature on the application and ethical challenges of AI in specific nursing contexts (1, 2).

### 4.4 Cognitive pathway differentiation: “novice-expert” differences in ethics and utility

This study further reveals the differences in core cognitive nodes between novices and experts.

1. Nurse Interns (Novices): Over-centralization of Ethics and Utility Cognition. The nurse intern group’s centrality was significantly higher than that of the clinical preceptor group on nodes such as ET_1 (Ethical compliance) and ET_3 (AI misuse vigilance), as well as on ethics-related bridge nodes (ET_1 Ethical compliance, ET_2 Privacy/security negligence). This indicates that, as “digital natives” (15), this new generation of nurses is highly sensitive to the ethical, privacy, and security issues (ET_1, ET_2) brought about by digital technology. These abstract ethical considerations and the vague cognition of AI’s utility (AW_2 AI utility unawareness) are placed in an over-centralized position within their “undifferentiated” cognitive network (node strength centrality see Figure 3C, bridge strength centrality see Figure 4), becoming the primary “transfer station” for their AI perception.
2. Clinical Preceptors (Experts): The Integrated Path of Application-Ethics. Clinical preceptors’ node strength in “EV_3 (Context-specific AI adoption)” was significantly higher than that of the interns (Figure 3C). As previously mentioned, their network is characterized by the strong integration of “application” and “ethics.” This suggests that experts’ cognition has moved beyond generalized discussions of “what AI is” and has entered a concrete assessment phase of “how to use AI safely within specific processes.” This reflects the experts’ cognitive characteristic of focusing more on placing AI evaluation within specific clinical workflows.

### 4.5 Implications for public health policy, management, and education

The network analysis results of this study, namely the dominant core position of “AI Fear” (N_3 Technological dread association, N_6 Future application anxiety) in the cognitive network, and the fundamental differences in cognitive structure between novices (high-density, undifferentiated network) and experts (low-density, strong-structure network), provide key implications for nursing education and clinical AI promotion. The study clearly indicates that “one-size-fits-all” AI training is destined to be inefficient because it fails to address the core cognitive barriers of specific groups. Future AI education and clinical integration must shift from “training isolated skills” to “reshaping the entire cognitive network” and must provide precise, theoretically-grounded intervention pathways for nurses at different experience levels.

1. Intervening in Core Fear (N_3/N_6): From Reassurance to Cognitive Restructuring.

Any AI education’s primary task must be to address the two high-intensity core nodes, N_3 (Technological dread association) and N_6 (Future application anxiety). If this “organizing hub” of fear is not shaken, any skill training will be only half as effective.

·Beyond Simple Reassurance that “AI is an Assistant”: Simply informing nurses that AI is an “assistant” rather than a “substitute” is insufficient (13). This fear is rooted in a sense of threat to professional identity and value (8). Therefore, educational interventions must delve into the Affective Domain. Educators should recognize that AI training must transcend operational skills to address the emotional adaptation of nursing personnel. For instance, “Reflective Critical Thinking Workshops” could be introduced to guide nurses in discussing potential professional identity crises or concerns regarding the erosion of “humanistic nursing care” brought about by AI integration, thereby establishing a rational perception of technology at the affective level. Furthermore, role-playing activities themed around “Human-AI Collaborative Ethics” can be conducted, allowing nursing staff to experience and manage responsibility-related anxiety and trust-building during AI-assisted decision-making in simulated scenarios. These interventions aim to facilitate their transition from “technological fear” to “emotional acceptance.”

Applying “Transformative Learning Theory”: Educators should draw on Mezirow’s “Transformative Learning” theory (31), designing AI training as a “transformative learning” experience. According to Mezirow’s theory, this first requires the creation of “Disorienting Dilemmas.” For example, in contemporary nursing training, case studies can be used to show how AI detected early signs of sepsis that human nurses missed. Nurses are then guided to “critically reflect” on their original “AI threat” assumptions. This reflection can help them restructure their cognitive framework, viewing AI as a tool that enhances their professional judgment and patient care capabilities.

Fostering a “Psychologically Safe” Training Environment: Managers should provide a “low-risk, high-support” learning environment for nursing staff, particularly for novices in the cognitive construction phase. This environment allows them to safely express their fears, concerns, and seemingly “elementary” questions without fear of judgment, which is a prerequisite for achieving cognitive restructuring (32). For instance, “AI Simulation Workshops” could be implemented, allowing nurse interns to practice within simulation systems (such as virtual labor monitoring systems) that do not involve actual patient safety. In such a setting, operational errors do not result in adverse clinical consequences; this “permission to fail” mechanism can effectively alleviate technological apprehension among novices. Furthermore, establishing “peer support groups” where experienced clinical preceptors serve as mentors to share their own frustrations and experiences in overcoming challenges while adapting to AI technology can reduce self-doubt among novices through psychological resonance.

1. Activating Key Bridges (US_3/ET_1): Integrating Simulation with Ethical Reflection.

Our study found that US_3 (Workflow efficiency enhancement) and ET_1 (Ethical compliance) are the strongest bridges connecting the two major communities of “Literacy” and “Attitude.” Therefore, educational curricula must be designed around these two key bridge nodes to ensure technology acceptance.

·US_3 (Workflow efficiency enhancement): From “Telling” to “Demonstrating.” Nurses’ (especially experienced experts’) acceptance of AI is highly dependent on its perceived relative advantage and practical value (3).

Applying High-Fidelity and *In-Situ* Simulation (Simulation): Rather than using PPT lectures, high-fidelity simulation cases should be developed. For example, having nurses use an algorithm-driven clinical decision support (CDSS)—specifically an Early Warning Scoring system (EWS)—within a simulated Electronic Health Record (EHR) system to manage a rapidly deteriorating simulated patient. Research has confirmed that this type of simulation-based training can significantly improve nurses’ professional competence and self-confidence in using EWS tools, helping them better identify patient deterioration (33).

ET_1 (Ethical compliance): From “Rules” to “Reasoning.” Ethics (ET_1) is another core bridge, with particularly high centrality in the novice network. This indicates that education must move beyond the level of merely “following rules” (11).

Applying “Reflective Practice” and “Case-Based Methods”: To ensure the depth and durability of AI ethics education, educators should integrate it as a longitudinal curriculum, rather than a one-off lecture, as the latter is unlikely to bring about profound professional transformation (34). This curriculum should adopt Problem-Based Learning (PBL) (35) and ethical vignettes as teaching methods, guiding nurses to proactively analyze the complex challenges posed by AI, such as algorithmic bias, health data privacy and confidentiality, and other key issues (36). More importantly, Schön’s theory of “Reflective Practice” (37) should be deeply integrated. Educators should guide nurses to write reflective journals or create digital portfolios to deeply explore the ethical dilemmas and internal conflicts brought about by AI in ambiguous clinical contexts (38). This continuous, guided reflection is the core pathway to cultivating nurses’ advanced ethical reasoning skills and “Digital Professionalism” (39).

1. Implementing Stratified Educational Strategies (Novice vs. Expert): Precision Intervention Based on Cognitive Networks.

The most central finding of this study is the structural difference between novice and expert cognitive networks. Educational strategies must be matched to this cognitive structure.

For Nurse Interns (Novices): Provide “Structured Scaffolding.” The novices’ “high-density, undifferentiated” network indicates their cognition is diffuse, easily disturbed, and that ethical nodes (ET_1 Ethical compliance, ET_3 AI misuse vigilance) are over-centralized. They require structure and guidance.

Strategy: Apply Scaffolding Theory (22).

Step 1: Establish an “AI Literacy” foundation. First, provide a clear, standardized AI literacy competency framework that clearly defines “what is AI,” “what is algorithmic bias,” and “the basic capabilities and limitations of AI.” This provides a stable “cognitive anchor” for their chaotic network.

Step 2: Provide “decontextualized” rules. Consistent with the “Novice to Expert” nursing practice theoretical framework (14), novices require clear, specific rules. Teaching should begin with low-risk, structured AI applications (such as AI-assisted scheduling or documentation) before gradually transitioning to high-risk CDSS. This helps them “prune” the network, establishing rational connections between abstract ethical fears (ET_1 Ethical compliance, ET_3 AI misuse vigilance) and concrete, manageable application scenarios (US_1 AI-assisted task execution, EV_3 Context-specific AI adoption).

For Clinical Preceptors (Experts): Cultivating “AI Champions” and “Evaluation Capability.” The experts’ “low-density, strong-structure” network indicates their cognition is differentiated and resilient. Their strength in EV_3 (Context-specific AI adoption) is higher, and their “application-safety” pathways (e.g., US_1 (AI-assisted task execution)—ET_2 (Privacy/security negligence), EV_3 (Context-specific AI adoption)—ET_2 (Privacy/security negligence)) are more strongly connected.

Strategy: Adopt a “Train-the-Trainer” (TTT) model (23) to cultivate them as frontline “AI Champions” (40).

Step 1: Strengthen “Evidence-based” Evaluation Capability. The training focus for experts should not be “how to use,” but “how to evaluate” (EV_3 Context-specific AI adoption). To practice the “E” (Evidence-based) pillar of the “IDEA” framework proposed by Teferi (41), the curriculum should focus on Evidence-Based Practice (EBP) for technology assessment. This enables nurses to Critically Appraise the effectiveness, reliability, and bias risk of AI tools, and their suitability for specific clinical workflows.

Step 2: Empower “Leadership and Translation” Roles. Leveraging their established “application-safety” cognitive pathways (US_1 (AI-assisted task execution)—ET_2 (Privacy/security negligence)), they should be trained to lead AI translation and implementation science in clinical practice. Their role is to act as “clinical context translators,” translating AI technical language into nurses’ clinical language, and to guide and support novice nurses, thereby creating a sustainable culture of technology adoption and learning within the department.

From a public health management perspective, these findings suggest that digital health integration requires more than technical training. Health authorities should prioritize “psychological safety” and “cognitive scaffolding” within organizational policies to reduce systemic technology-related anxiety and ensure the sustainability of AI-driven healthcare services.

## 5 Limitations

Although this study is the first to use PNA to reveal the “novice-expert” differences in AI cognition among maternal and child nurses, the following limitations must be considered, and caution should be exercised when interpreting the results:

1. Design Limitations: This study utilized a cross-sectional design, collecting data from two groups, clinical preceptors and nurse interns, at the same time point (January to March 2025). While this design can effectively reveal significant differences in AI cognitive network structures between the two groups (e.g., the “high-density” network of interns vs. the “strong-structure” network of preceptors), it cannot provide evidence regarding causal relationships or developmental trajectories. We cannot conclude that the network structure of nurse interns (novices) will necessarily “evolve” into the cognitive structure of clinical preceptors (experts) with the accumulation of clinical experience. This observed difference may also stem from generational effects (such as the characteristics of “digital natives”) or other unmeasured confounding factors.
2. Data Limitations: The data in this study relied entirely on self-report scales, namely the AILS and GAAIS. Although the study used anonymous surveys to reduce apprehension, social desirability bias remains a risk that cannot be entirely excluded. When participants (especially nurses) answered sensitive items related to “AI fear” (such as N_3 “Technological dread association”) or “ethical compliance” (such as ET_1 “Ethical compliance”), they may have been inclined to provide answers more consistent with their professional image or social expectations, rather than their true internal attitudes. This bias might affect the strength estimation of core nodes in the network (especially those related to “fear”) or underestimate the true prevalence of certain negative attitudes.
3. Sampling Limitations: This study’s sampling strategy has dual limitations. First, the study used convenience sampling to recruit subjects, which means the sample may not fully represent the nurse population in all MCH institutions in China, and selection bias may exist (i.e., nurses who are more interested in or more anxious about AI may have been more willing to participate). Second, the sample is highly concentrated in a specific professional field—maternal and child specialty nurses (including obstetrics, gynecology, and pediatrics). The work characteristics of the maternal and child nursing field (such as high emotional involvement and care for vulnerable populations) may have shaped this group’s unique cognition of AI (especially the fear of “dehumanization”). Therefore, whether the cognitive network structure found in this study (especially the core status of “AI Fear”) can be generalized to other nursing specialties (such as emergency, surgical, or geriatric nursing) and to other cultural backgrounds outside of mainland China requires further verification. While the inclusion of 26 provinces is a strength, future research should account for regional disparities in public health infrastructure and digital development levels across China.

## 6 Future directions

The findings and limitations of this study provide two key directions for the future research agenda, aiming to deepen the understanding of AI cognitive networks from the perspectives of dynamic development and causal intervention:

1. Conduct Longitudinal Studies to verify the “Novice-to-Expert” network evolution: This study’s cross-sectional design revealed significant differences in cognitive network structures between “novices” and “experts,” but it cannot confirm if this is a process that evolves with growing experience. A primary future task is to conduct a prospective longitudinal study. This would involve recruiting a cohort of nurse interns (Novices) about to graduate and conducting repeated measurements at key junctures in their careers (e.g., at Year 1, Year 3, and Year 5 post-employment) to observe and quantify whether the AI cognitive network of the same cohort “reorganizes” as clinical experience accumulates. This would verify if the network structure, as predicted by the “Novice to Expert” theory, gradually “Prunes” and “Strengthens” from a “high-density, undifferentiated” diffuse state, eventually evolving into the “low-density, strong-structure” expert network. This will help distinguish the different impacts of the “Experience effect” versus the “Generational effect” on AI cognitive structure.
2. Design Precision Intervention Studies based on network nodes: This study identified “core nodes” (e.g., N_3 “Technological dread association”) and “bridge nodes” (e.g., US_3 “Workflow efficiency enhancement”) within the cognitive network. The practical value of this finding urgently needs verification through intervention research. Future studies should shift from “one-size-fits-all” AI training models to designing precision intervention protocols “based on network nodes.” Modular interventions targeting specific nodes should be developed. For example, designing an intervention module based on “Transformative Learning” and “Psychological Safety” to specifically target core fear nodes (N_3 Technological dread association, N_6 Future application anxiety) for cognitive restructuring; simultaneously, designing a hands-on training module based on “high-fidelity simulation” to specifically activate and strengthen the two key bridges of “efficiency” (US_3 Workflow efficiency enhancement) and “ethics” (ET_1 Ethical compliance). Intervention studies should use PNA as the core assessment tool, conducting Randomized Controlled Trials (RCTs) or quasi-experimental designs. By comparing the changes in network structure between the intervention and control groups before and after the intervention (rather than just scale total scores), researchers can verify whether the intervention successfully “reshaped” the cognitive network (e.g., whether it significantly reduced the centrality of N_3 or strengthened the connection between US_3 and the positive attitude community). This would provide deeper, mechanistic evidence for the effectiveness of AI educational strategies.

## 7 Conclusion

This study, utilizing PNA for the first time, quantified the AI cognitive network of maternal and child nurses, finding that “AI Fear” (N_3 Technological dread association, N_6 Future application anxiety) is the dominant core of this network. More importantly, the research revealed a fundamental difference between the “high-density, undifferentiated” network of novices (nurse interns) and the “low-density, strong-structure” network of experts (clinical preceptors). This finding strongly calls out that “one-size-fits-all” AI promotion strategies are destined to fail. Future AI education and clinical integration should adopt an integrated approach that encompasses both continuous skills training and the reshaping of cognitive networks, providing precise, stratified intervention pathways for nurses at different experience levels to keep pace with the dynamic evolution of AI technologies.

## Data Availability

The original contributions presented in the study are included in the article/Supplementary material, further inquiries can be directed to the corresponding author.

## Footnotes

1
https://www.wjx.cn/
