# Supplementary material for: AI literacy and attitudes among maternal and child health nurses: a multicenter psychological network analysis of novices and experts in China
Source: Front Public Health. 2026 Jun 2;14:1786184. doi: 10.3389/fpubh.2026.1786184 (PMC13269257; doi:10.3389/fpubh.2026.1786184)
Supplement: Supplementary file 1 [file Supplementary_file_1.DOCX]

**Supplementary Materials**

Content

**Table S1.** Characteristics of the clinical preceptor and nurse intern. 1

**Table S2.** The artificial intelligence literacy scale**(AILS)** and general attitudes towards artificial intelligence scale (GAAIS) and descriptive statistics for each question within the scale. 3

**Table S3.** List of nodes, their predictability, and their centality estimation 5

**Table S4.** Edge weight difference test between identity level networks 7

**Figure S1.** Bootstrapped difference test of edge weights. 8

**Figure S2.** Bootstrapped difference test of the node strength centrality. 9

**Figure S3.** Bootstrapped difference test of the node bridge strength centrality 10

**Table 1 Characteristics of the clinical preceptor and nurse intern**

| Characteristics | Frequency / Percentage (*n* / %) | |
| --- | --- | --- |
|  | Clinical preceptor (*n*=498) | Nurse intern (*n*=533) |
| Gender |  |  |
| Male | 8（1.6） | 66（12.4） |
| Female | 490（98.4） | 467（87.6） |
| Age (years) |  |  |
| ≤30 / ≤18 ^a^ | 103（20.7） | 88（16.5） |
| 31 - 40 / 18 - 20 ^a^ | 294（59.0） | 145（27.2） |
| 41 - 50 / 20 - 22 ^a^ | 95（19.1） | 244（45.8） |
| ≥51 / ≥22 ^a^ | 6（1.2） | 56（10.5） |
| Ethnicity |  |  |
| Han | 456（91.6） | 471（88.4） |
| Others | 42（8.4） | 62（11.6） |
| Marital status ^b^ |  |  |
| Married | 434（87.1） | — |
| Single | 64（12.9） | — |
| Family type |  |  |
| Intact two-parent family | — | 445（83.5） |
| Reconstituted family | — | 27（5.1） |
| Single-parent family | — | 61（11.4） |
| Household registration location |  |  |
| Countryside | — | 368（69.0） |
| Town | — | 165（31.0） |
| Working experience (years) |  |  |
| ≤ 5 | 38（7.6） | — |
| 6 - 10 | 130（26.1） | — |
| 11 - 15 | 177（35.5） | — |
| ≥ 16 | 153（30.7） | — |
| Professional title |  |  |
| Junior | 155（31.1） | — |
| Intermediate | 288（57.8） | — |
| Senior | 55（11.0） | — |
| Current educational attainment |  |  |
| Associate degree or below | — | 358（67.2） |
| Bachelor's degree or higher | — | 175（32.8） |
| Work/Intern Department ^c^ |  |  |
| Obstetrics and gynecology | 121（24.3） | 223（41.8） |
| Pediatrics | 245（49.2） | 264（49.5） |
| Maternal and child health | 132（26.5） | 46（8.6） |
| Work/Intern Hospital type |  |  |
| General hospital | 271（54.4） | 316（59.3） |
| Specialized hospital | 227（45.6） | 217（40.7） |
| Work/Intern Hospital level ^d^ |  |  |
| Tertiary grade A | 302（60.6） | 359（67.4） |
| Tertiary grade B | 76（15.3） | 65（12.2） |
| Secondary grade | 120（24.1） | 109（20.5） |
| Training experience in AI |  |  |
| Received specialized training | 73（14.7） | 144（27.0） |
| No specialized training, but has been exposed to related topics or self-studied | 217（43.6） | 215（40.3） |
| No prior exposure to the topic | 208（41.8） | 174（32.6） |
| ***Note***. Dashes (-) indicate variables not applicable to the group.  ^a^ Age ranges differ between groups due to cohort characteristics (clinical preceptors vs. nurse interns).  ^b^ Single indicated separated, divorced, widowed, or never married, and married indicated married or partnered.  ^c^ In this study, maternal and child health nurses were categorized into three major departments based on their service recipients: **Obstetrics and Gynecology:** This department focuses on the care of pregnant women, postpartum women, and women’s reproductive health, encompassing prenatal, intrapartum, and postnatal care, as well as the management of gynecological conditions. **Pediatrics:** This department provides healthcare for children from newborns to adolescents, including disease prevention, vaccination, and monitoring of growth and development. **Maternal and Child Health:** This department integrates health promotion, disease prevention, and health education for both women and children.  ^d^ Hospital level indicates the hospital’s capacity and quality in China. Tertiary grade A: Top-tier hospitals with highest-level services and advanced technology. Tertiary grade B: Similar to grade A but slightly lower in capacity. Secondary grade: Mid-level facilities serving smaller regions. | | |

**Table S2.** The artificial intelligence literacy scale**(AILS)** and general attitudes towards artificial intelligence scale (GAAIS) and descriptive statistics for each question within the scale**.**

| **Construct** | **Item** | **Description** | **Mean±SD** | | **t** | ***P*** |
| --- | --- | --- | --- | --- | --- | --- |
|  |  |  | **Clinical preceptor (*N*=498)** | **Nurse intern (*N*=533)** |  |  |
| ****AILS**** | | | 56.10±9.50 | 54.94±8.23 | 2.100 | 0.036 |
| Awareness | AW_1 | I can distinguish between smart devices and non-smart devices | 4.88±1.20 | 4.92±1.23 | -0.497 | 0.619 |
|  | AW_2 | I do not know how AI technology can help me ^R^ | 4.11±1.45 | 3.70±1.29 | 4.741 | 0.000 |
|  | AW_3 | I can identify the AI technology employed in the applications and products I use | 4.73±1.19 | 4.74±1.20 | -0.165 | 0.869 |
| Usage | US_1 | I can skilfully use AI applications or products to help me with my daily work. | 4.49±1.29 | 4.64±1.25 | -1.901 | 0.058 |
|  | US_2 | It is usually hard for me to learn to use a new AI application or product ^R^ | 3.65±1.49 | 3.88±1.30 | -2.582 | 0.010 |
|  | US_3 | I can use AI applications or products to improve my work efficiency | 5.06±1.21 | 4.89±1.17 | 2.386 | 0.017 |
| Evaluation | EV_1 | I can evaluate the capabilities and limitations of an AI application or product after using it for a while | 4.95±1.18 | 4.78±1.18 | 2.226 | 0.026 |
|  | EV_2 | I can choose a proper solution from various solutions provided by a smart agent | 4.95±1.19 | 4.80±1.16 | 2.011 | 0.045 |
|  | EV_3 | I can choose the most appropriate AI application or product from a variety for a particular task | 4.99±1.13 | 4.83±1.14 | 2.248 | 0.025 |
| Ethics | ET_1 | I always comply with ethical principles when using AI applications or products | 5.48±1.17 | 5.09±1.21 | 5.151 | 0.000 |
|  | ET_2 | I am never alert to privacy and information security issues when using AI applications or products ^R^ | 3.85±1.64 | 3.85±1.42 | 0.056 | 0.955 |
|  | ET_3 | I am always alert to the abuse of AI technology | 4.96±1.27 | 4.82±1.25 | 1.885 | 0.060 |
| **GAAIS** | | | 70.51±10.62 | 66.61±8.85 | 6.382 | 0.000 |
| Positive attitude | P_1 | For routine transactions, I would rather interact with an artificially intelligent system than with a human | 3.16±0.99 | 3.15±1.01 | 0.173 | 0.862 |
|  | P_2 | Artificial Intelligence can provide new economic opportunities for this country | 3.85±0.78 | 3.71±0.81 | 2.915 | 0.004 |
|  | P_3 | Artificially intelligent systems can help people feel happier | 3.67±0.77 | 3.55±0.80 | 2.389 | 0.017 |
|  | P_4 | I am impressed by what Artificial Intelligence can do | 3.61±0.78 | 3.46±0.82 | 2.974 | 0.003 |
|  | P_5 | I am interested in using artificially intelligent systems in my daily life | 3.86±0.74 | 3.67±0.80 | 3.962 | 0.000 |
|  | P_6 | Artificial Intelligence can have positive impacts on people's wellbeing | 3.78±0.77 | 3.6±0.80 | 3.772 | 0.000 |
|  | P_7 | Artificial Intelligence is exciting | 3.61±0.79 | 3.43±0.79 | 3.642 | 0.000 |
|  | P_8 | An artificially intelligent agent would be better than an employee in many routine jobs | 3.42±0.90 | 3.25±0.89 | 3.091 | 0.002 |
|  | P_9 | There are many beneficial applications of Artificial Intelligence | 3.84±0.70 | 3.66±0.76 | 3.963 | 0.000 |
|  | P_10 | Artificially intelligent systems can perform better than humans | 3.41±0.84 | 3.27±0.87 | 2.685 | 0.007 |
|  | P_11 | Much of society will benefit from a future full of Artificial Intelligence | 3.73±0.75 | 3.51±0.81 | 4.517 | 0.000 |
|  | P_12 | I would like to use Artificial Intelligence in my own job | 3.78±0.76 | 3.56±0.81 | 4.467 | 0.000 |
| Negative attitude | N_1 | Organisations use Artificial Intelligence unethically | 3.24±1.10 | 3.07±1.01 | 2.638 | 0.008 |
|  | N_2 | I think artificially intelligent systems make many errors | 3.16±0.89 | 2.98±0.86 | 3.294 | 0.001 |
|  | N_3 | I find Artificial Intelligence sinister | 3.51±1.05 | 3.21±0.97 | 4.822 | 0.000 |
|  | N_4 | Artificial Intelligence might take control of people | 3.35±1.04 | 3.16±1.00 | 3.013 | 0.003 |
|  | N_5 | I think Artificial Intelligence is dangerous | 3.32±0.98 | 3.06±0.90 | 4.521 | 0.000 |
|  | N_6 | I shiver with discomfort when I think about future uses of Artificial Intelligence | 3.46±1.02 | 3.18±0.97 | 4.565 | 0.000 |
|  | N_7 | People like me will suffer if Artificial Intelligence is used more and more | 3.45±1.00 | 3.09±0.95 | 5.890 | 0.000 |
|  | N_8 | Artificial Intelligence is used to spy on people | 3.29±1.05 | 3.05±0.97 | 3.800 | 0.000 |
| *Note:* R:Indicates reverse-scored item; AW：Awareness, US: Usage, EV: Evaluation, ET: Ethics; P:Positive attitude, N: Negative attitude | | | | | | |

**Table S3.** List of nodes, their predictability, and their centality estimation

| **Nodes** | **Variables** | **Strength** | **BridgeStrength** | **ExpectedInfluence** | **Bridge Expected Influence (1-step)** | **Predictability (R^2^)** |
| --- | --- | --- | --- | --- | --- | --- |
| AW_1 | Smart device identification | -1.363 | 0.433 | -0.780 | 0.679 | 0.511 |
| AW_2 | AI utility unawareness | -1.865 | 1.085 | -1.531 | 1.101 | 0.418 |
| AW_3 | AI technology recognition | 0.387 | 1.428 | 0.751 | 1.689 | 0.712 |
| US_1 | AI-assisted task execution | -0.046 | 1.673 | -0.375 | 1.422 | 0.671 |
| US_2 | AI tool learning difficulty | -0.576 | 1.821 | -0.835 | 1.556 | 0.458 |
| US_3 | Workflow efficiency enhancement | 0.715 | 2.102 | 0.721 | 2.150 | 0.741 |
| EV_1 | Capability/limitation assessment | 0.228 | 0.829 | 0.120 | 0.769 | 0.720 |
| EV_2 | Optimal solution selection | 0.704 | 0.391 | 0.892 | 0.585 | 0.768 |
| EV_3 | Context-specific AI adoption | 0.746 | 1.069 | 1.033 | 1.324 | 0.724 |
| ET_1 | Ethical compliance | 1.197 | 1.948 | 0.645 | 1.680 | 0.638 |
| ET_2 | Privacy/security negligence | -2.560 | 0.701 | -1.811 | 0.907 | 0.293 |
| ET_3 | AI misuse vigilance | -1.571 | 0.386 | -1.820 | 0.042 | 0.458 |
| P_1 | Routine interaction preference | -1.090 | -0.114 | -2.433 | -1.125 | 0.426 |
| P_2 | Economic opportunity creation | -0.614 | -0.592 | -0.085 | -0.299 | 0.644 |
| P_3 | Subjective well-being boost | -0.076 | -1.001 | 0.365 | -0.702 | 0.710 |
| P_4 | Technical capability recognition | 0.390 | -0.735 | 0.627 | -0.524 | 0.689 |
| P_5 | Daily application propensity | 0.368 | -0.717 | 0.735 | -0.422 | 0.739 |
| P_6 | Welfare enhancement potential | 0.631 | -0.933 | 0.882 | -0.684 | 0.770 |
| P_7 | Technological enthusiasm | 0.491 | -0.832 | 0.599 | -0.694 | 0.758 |
| P_8 | Routine task superiority | 0.178 | -0.758 | -0.259 | -1.019 | 0.652 |
| P_9 | Application diversity | 0.586 | -0.308 | 0.918 | -0.019 | 0.731 |
| P_10 | Human-AI performance gap | -0.007 | -0.749 | -0.258 | -0.906 | 0.663 |
| P_11 | Societal benefit anticipation | 0.629 | -0.720 | 0.854 | -0.491 | 0.753 |
| P_12 | Professional adoption intent | 0.609 | -0.791 | 0.937 | -0.496 | 0.757 |
| N_1 | Ethical misconduct applications | -1.431 | -0.734 | -1.676 | -1.042 | 0.560 |
| N_2 | Systemic error perception | -0.680 | -0.872 | -0.641 | -0.907 | 0.690 |
| N_3 | Technological dread association | 1.978 | -0.210 | 0.908 | -0.702 | 0.796 |
| N_4 | Control risk anticipation | -0.090 | -0.883 | -0.016 | -0.830 | 0.751 |
| N_5 | Safety hazard awareness | 0.430 | -0.834 | 0.303 | -0.859 | 0.809 |
| N_6 | Future application anxiety | 1.484 | -0.601 | 0.999 | -0.752 | 0.861 |
| N_7 | Collective welfare threat | 0.670 | -0.806 | 0.942 | -0.540 | 0.821 |
| N_8 | Privacy surveillance concern | -0.453 | -0.678 | -0.714 | -0.891 | 0.699 |
| *Note:* AW：Awareness, US: Usage, EV: Evaluation, ET: Ethics; P:Positive attitude, N: Negative attitude. | | | | | | |

**Table S4.** Edge weight difference test between identity level networks

| **Var1: Clinical preceptor** | **Var2: Nurse intern** | ***P*-value** | **Test statistic E** |
| --- | --- | --- | --- |
| AW_1 Smart device identification | EV_1 Capability/limitation assessment | 0.045 | 0.120 |
| AW_3 AI technology recognition | ET_2 Privacy/security negligence | 0.010 | 0.030 |
| US_1 AI-assisted task execution | ET_2 Privacy/security negligence | 0.001 | 0.279 |
| EV_2 AI tool learning difficulty | ET_2 Privacy/security negligence | 0.001 | 0.086 |
| EV_3 Context-specific AI adoption | ET_2 Privacy/security negligence | 0.001 | 0.161 |
| ET_1 Ethical compliance | ET_2 Privacy/security negligence | 0.001 | 0.156 |
| AW_2 AI utility unawareness | ET_3 AI misuse vigilance | 0.001 | 0.189 |
| US_2 AI tool learning difficulty | ET_3 AI misuse vigilance | 0.044 | 0.020 |
| AW_2 AI utility unawareness | P_1 Routine interaction preference | 0.002 | 0.061 |
| US_2 AI tool learning difficulty | P_1 Routine interaction preference | 0.003 | 0.070 |
| AW_2 AI utility unawareness | P_3 Subjective well-being boost | 0.028 | 0.010 |
| EV_1 Capability/limitation assessment | P_3 Subjective well-being boost | 0.047 | 0.001 |
| AW_1 Smart device identification | P_4 Technical capability recognition | 0.020 | 0.081 |
| AW_1 Smart device identification | P_5 Daily application propensity | 0.006 | 0.071 |
| ET_2 Privacy/security negligence | P_5 Daily application propensity | 0.004 | 0.017 |
| ET_1 Ethical compliance | P_6 Welfare enhancement potential | 0.044 | 0.014 |
| ET_2 Privacy/security negligence | P_7 Technological enthusiasm | 0.008 | 0.037 |
| P_2 Economic opportunity creation | P_7 Technological enthusiasm | 0.009 | 0.080 |
| US_1 AI-assisted task execution | P_8 Routine task superiority | 0.039 | 0.041 |
| ET_1 Ethical compliance | P_8 Routine task superiority | 0.034 | 0.066 |
| P_1 Routine interaction preference | P_8 Routine task superiority | 0.045 | 0.138 |
| ET_2 Privacy/security negligence | P_9 Application diversity | 0.014 | 0.016 |
| P_6 Welfare enhancement potential | P_9 Application diversity | 0.029 | 0.158 |
| EV_1 Capability/limitation assessment | P_10 Human-AI performance gap | 0.037 | 0.007 |
| AW_1 Smart device identification | EV_1 Capability/limitation assessment | 0.045 | 0.120 |

**
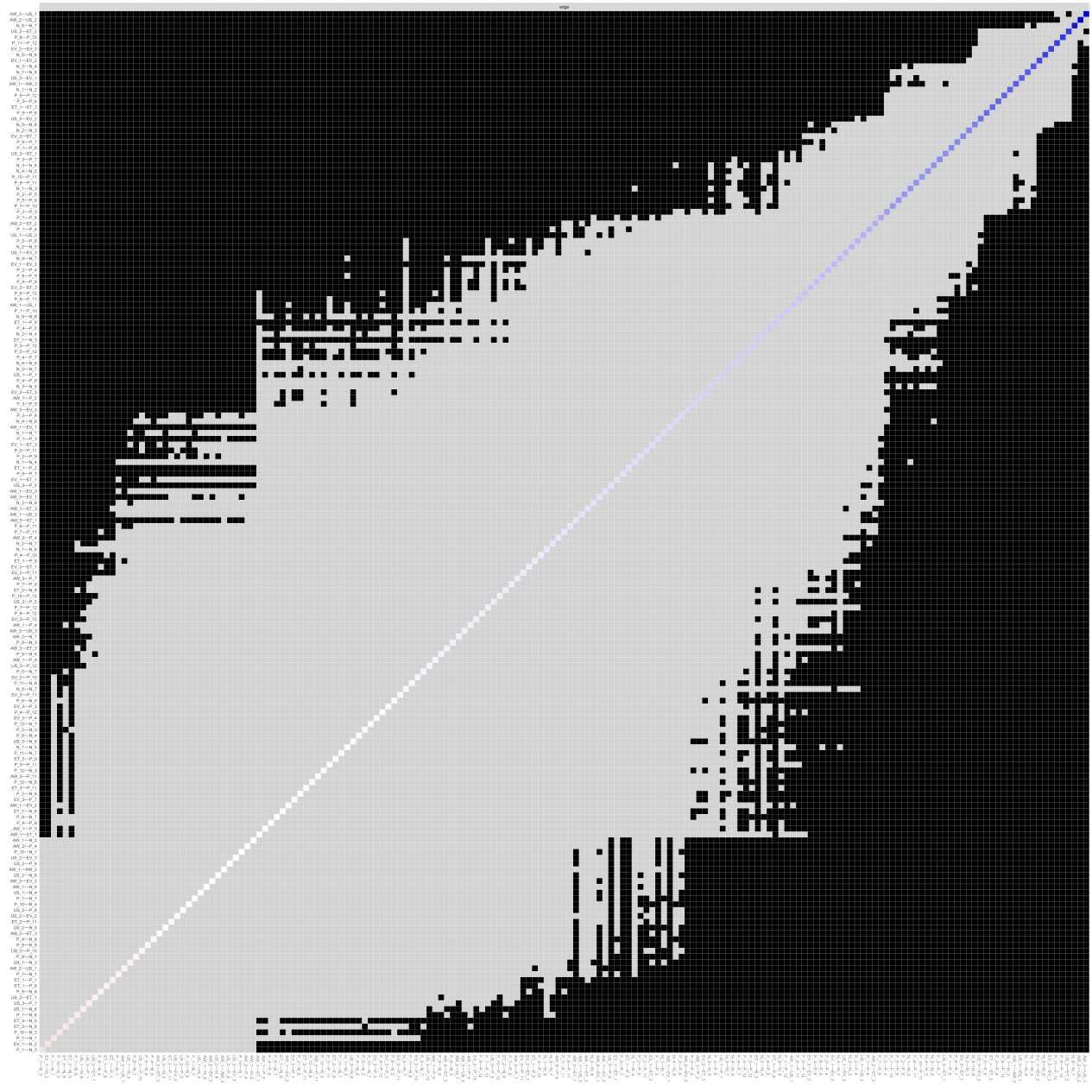
**

**Figure S1.** Bootstrapped difference test of edge weights.

*Note:* The x-axis and y-axis represent individual edge within the AILS and GAAIS network. Gray boxes indicate non-significant differences, while black boxes indicate significant differences.

.

**
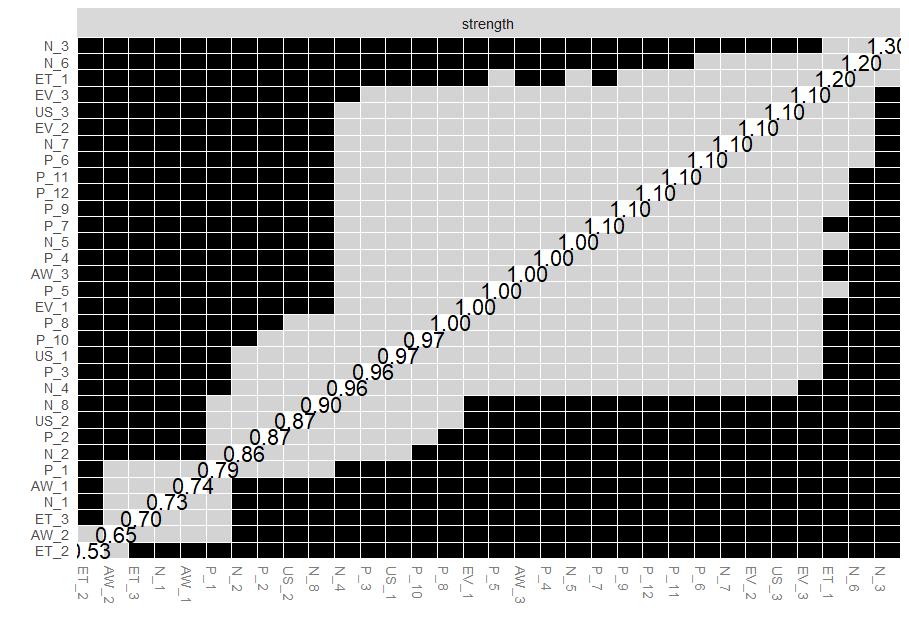
**

**Figure S2.** Bootstrapped difference test of the node strength centrality.

*Note:* The x-axis and y-axis represent individual nodes within the AILS and GAAIS network. Strength centrality values are plotted on the diagonal. Gray boxes indicate non-significant differences, while black boxes indicate significant differences.

**
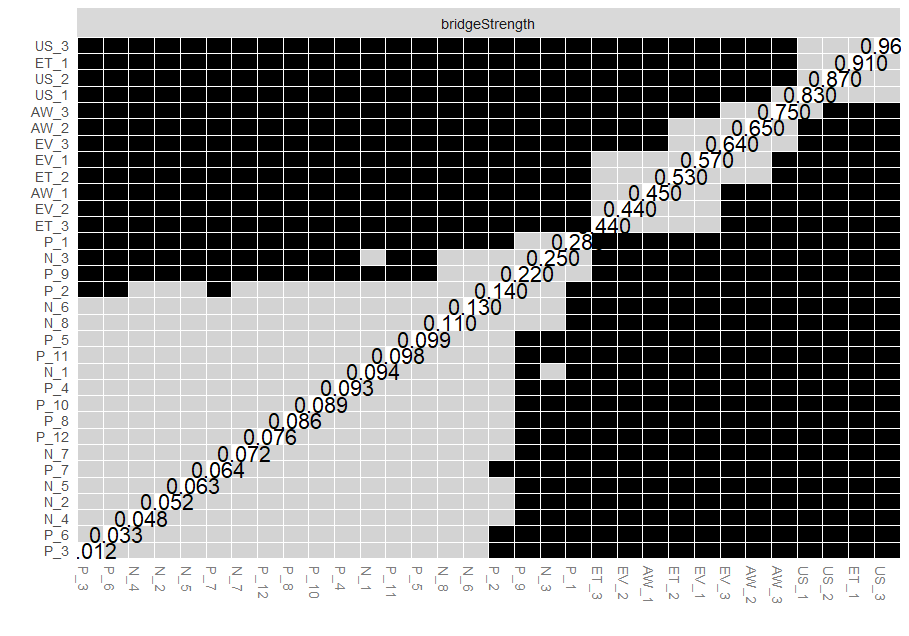
**

**Figure S3.** Bootstrapped difference test of the node bridge strength centrality.

*Note:* The x-axis and y-axis represent individual nodes within the AILS and GAAIS network. Node bridge strength values are plotted on the diagonal. Gray boxes indicate non-significant differences, while black boxes indicate significant differences.
